# 

**Published:** 1885-02

**Authors:** 


### Meeting.

The Forty-first Annual Meeting of the Mississippi Valley Association of Dental Surgeons will be held in the Lecture Room of the Ohio College of Dental Surgery on Wednesday, March 4th, 1885, at 10 o'clock A. M.

This is one of the first dental societies organized in this country, and, really, in the world; it is the oldest of those that have had a continuous existence. We account it dear because of its age and experience; because of its early and many associations; because of the many grand and noble men who have been of its membership; because of the many good and valuable things that have eminated from it, and because of the many important lessons of scientific and professional truth received from it.

It still has active and vigorous life enough, at least it is hoped for another forty years existence and work.

Its membership is not drawn from as large a territory as formerly; but there is as much—yes more—material from which to draw its members than in the early periods of its history, and so it should not want for support.

For the approaching meeting a good programme has been prepared, and is as follows:

1.—Progress of Dental Education.

2.—Recent Theories Concerning Caries of the Teeth.

3.—New Remedies for the Treatment of Oral and Dental Diseases.

4.—Improvements in Prosthetic Dentistry.

5.—New Methods and Materials for Filling Teeth.

6.—Incidents of Office Practice.

Officers.—Wm. Van Antwerp, President; F. W. Sage, First Vice President; A. F. Emminger, Second Vice President; A. G. Rose, Recording Secretary; F. A. Hunter, Treasurer; A. Berry, Corresponding Secretary.

Standing Committees.—Executive: E. G. Betty, C. R. Taft, and N. S. Hoff. Membership: F. A. Hunter, J. S. Cassidy, and A. G. Rose. Publication: J. Taft, J. M. Clyde, and O. N. Heise. Ethics: G. W. Keely, H. A. Smith, and C. M. Wright.

It is desirable that every member will come prepared to take some part in the work, with the view of making an interesting and profitable meeting.

### College Association.

The annual meeting of the stockholders of the Ohio College of Dental Surgery, will be held in the Lecture Room of the College, on College St., on Tuesday, March 3d, at 2 o'clock P. M.

It is desirable that all members be present, so far as possible, and give their aid and influence in promoting the cause of professional education.

### Alumni Meeting.

The annual meeting of the Alumni Association of the Ohio College of Dental Surgery will be held in the Lecture Room of the College, on Wednesday, P. M., March 4th. It is hoped that the Alumni will be largely present, and that every class will be represented as fully as possible, that old associations may be renewed, and friendships continued.

### Commencement.

The annual commencement exercises of the Ohio College of Dental Surgery will be held in College Hall on Walnut Street, between Fourth and Fifth Streets, on Wednesday, March 4th, at 8 o'clock P. M. The address on behalf of the trustees will be given by Dr. W. Storer Howe, of Philadelphia, and for the faculty by Prof. C. M. Wright. All friends of the College, and those interested, are cordially invited to be present.

### Married.

November 19th, Mr. Eugene Monroe Fay, of Hamilton, and Miss Mary Hall Keely, daughter of Dr. and Mrs. Geo. W. Keely, of Oxford, O.

By this, and another notice, it will be seen that Dr. K.'s family is growing rapidly, and that the glory of his house is increasing.

Everybody who knows the Dr.--and who does not?--will rejoice with him and his loved ones. May the joy and prosperity of these new families be as marked and as full as has been that of the patriarch of the family.

### CONTENTS.

### COMMUNICATIONS.

Married ........108

---

### THE DENTAL REGISTER,

**A MONTHLY MAGAZINE DEVOTED TO THE INTERESTS OF THE**

### DENTAL PROFESSION.

*Terms Reduced to $2.00 Per Year. Single Copies, 25 Cents.*

**EDITED BY PROF. J. TAFT, D.D.S.**

—AND—

**Published by SPENCER & CROCKER, Ohio Dental and Surgical Depot.**

The volume commences in January and closes in December.

The REGISTER being issued monthly is always fresh, therefore Indispensable to the practitioner who desires to keep posted up with the new inventions and improvements which are daily developed. Neither expense nor effort will be spared to give value and variety to its contents. Articles will be illustrated with well executed engravings whenever they will add to the interest or assist to explanation.

Its extensive circulation and frequency of issue make it one of the BEST DENTAL ADVERTISING MEDIUMS in the United States.

All subscriptions must begin with January or July.

Local or special notices, five lines or less, will be inserted for $3 each insertion; over five lines, 50 cts. per line.

All advertising bills payable in advance, or at furthest, after the issue of the first number containing the advertisement. All transient advertisements to be paid for in advance.

☞CONTRIBUTIONS SOLICITED.

Exclusively Editorial Communications should be addressed to the Editor.

The latest number of the REGISTER may be ordered with or without other goods at any time, and all letters should be addressed to

